# Supplementary material for: A yeast phenomic model for the influence of Warburg metabolism on genetic buffering of doxorubicin
Source: Cancer Metab. 2019 Oct 23;7:9. doi: 10.1186/s40170-019-0201-3 (PMC6806529; doi:10.1186/s40170-019-0201-3)
Supplement: Supplementary file 9 — Additional file 9. GO term-specific heatmaps for REMc/GTF-enriched clusters. GO term-specific heatmaps for significant GO process terms were generated as described in methods and Figs. 3 and 4. Any related child terms are presented in subsequent pages of the parent file name. GO terms with more than 100 children, with 2 or fewer genes annotated to the term, or a file size over 300KB are not shown. All heatmaps are generated with the same layout (see Figs. 3 and 4). [file 40170_2019_201_MOESM9_ESM.bz2 › Additional_File9_GOTermHeatmaps/Additional_File9_GOTermHeatmap/reciprocal_DNA_recombination.pdf]

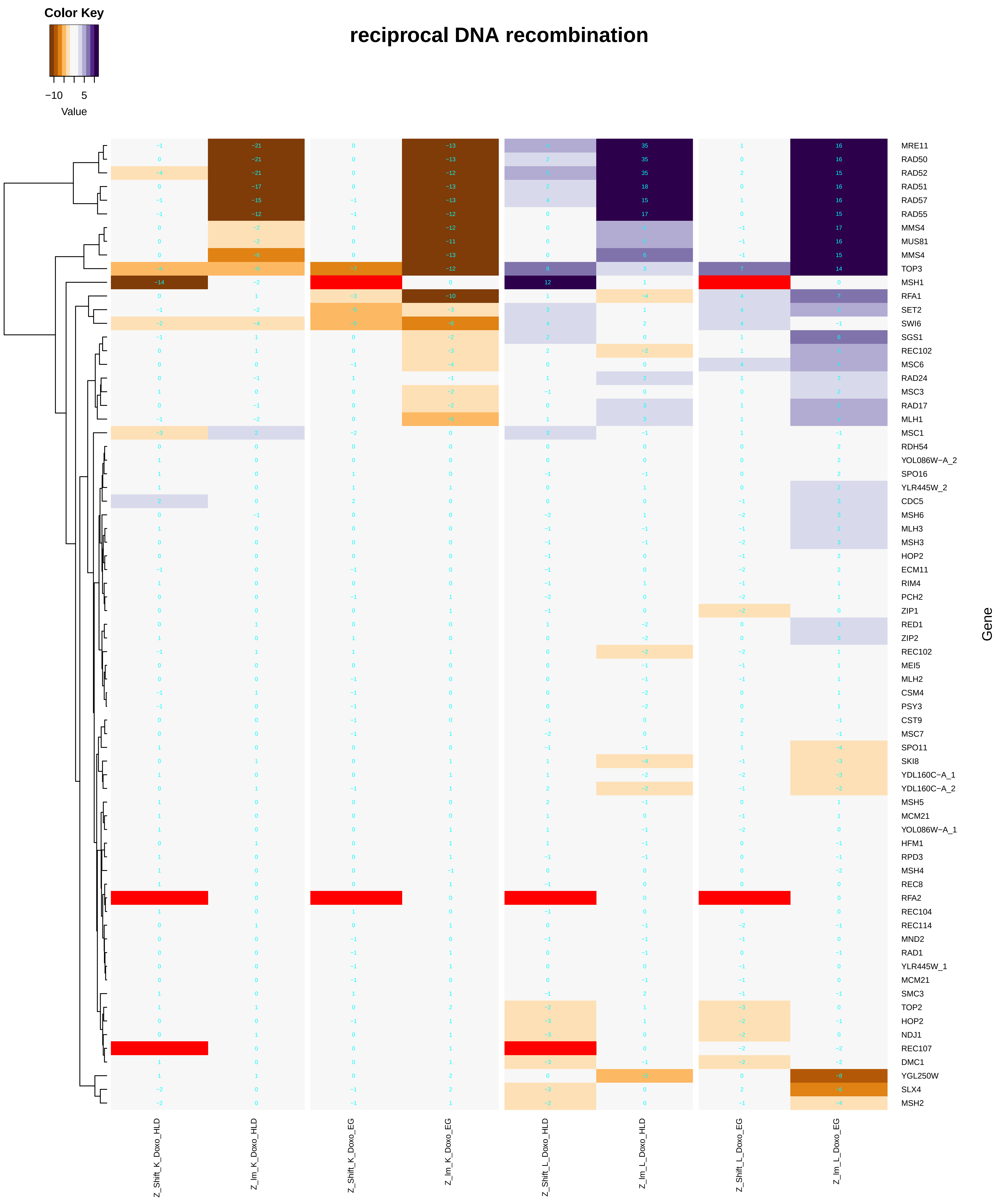

Color Key
−10
5
Value
reciprocal DNA recombination
MRE11
RAD50
RAD52
RAD51
RAD57
RAD55
MMS4
MUS81
MMS4
TOP3
MSH1
RFA1
SET2
SWI6
SGS1
REC102
MSC6
RAD24
MSC3
RAD17
MLH1
MSC1
RDH54
YOL086W−A_2
SPO16
YLR445W_2
CDC5
MSH6
MLH3
MSH3
HOP2
ECM11
RIM4
PCH2
ZIP1
RED1
ZIP2
REC102
MEI5
MLH2
CSM4
PSY3
CST9
MSC7
SPO11
SKI8
YDL160C−A_1
YDL160C−A_2
MSH5
MCM21
YOL086W−A_1
HFM1
RPD3
MSH4
REC8
RFA2
REC104
REC114
MND2
RAD1
YLR445W_1
MCM21
SMC3
TOP2
HOP2
NDJ1
REC107
DMC1
YGL250W
SLX4
MSH2
Gene
Z_Shift_K_Doxo_HLD
Z_lm_K_Doxo_HLD
Z_Shift_K_Doxo_EG
Z_lm_K_Doxo_EG
Z_Shift_L_Doxo_HLD
Z_lm_L_Doxo_HLD
Z_Shift_L_Doxo_EG
Z_lm_L_Doxo_EG

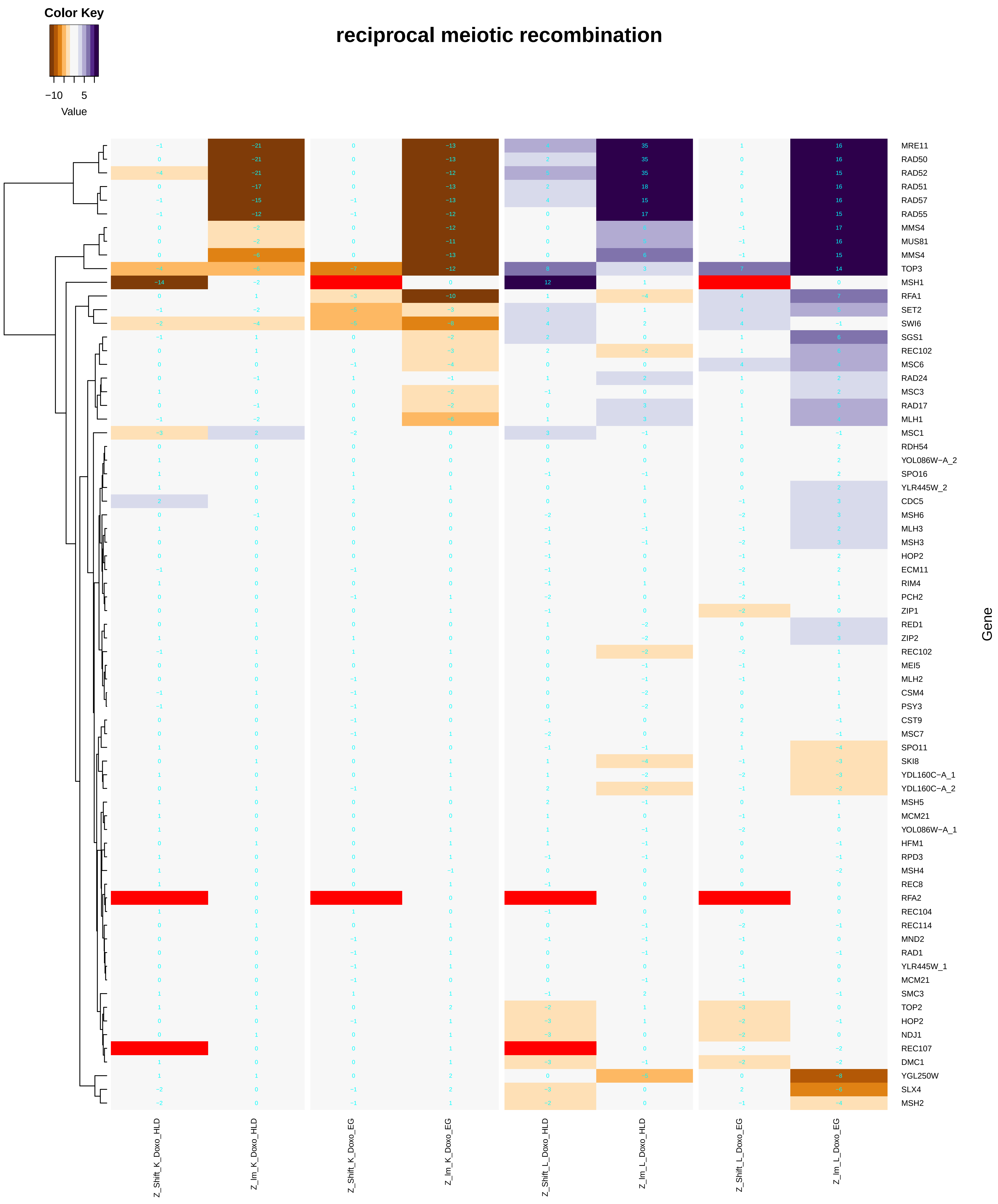

Color Key
−10
5
Value
reciprocal meiotic recombination
MRE11
RAD50
RAD52
RAD51
RAD57
RAD55
MMS4
MUS81
MMS4
TOP3
MSH1
RFA1
SET2
SWI6
SGS1
REC102
MSC6
RAD24
MSC3
RAD17
MLH1
MSC1
RDH54
YOL086W−A_2
SPO16
YLR445W_2
CDC5
MSH6
MLH3
MSH3
HOP2
ECM11
RIM4
PCH2
ZIP1
RED1
ZIP2
REC102
MEI5
MLH2
CSM4
PSY3
CST9
MSC7
SPO11
SKI8
YDL160C−A_1
YDL160C−A_2
MSH5
MCM21
YOL086W−A_1
HFM1
RPD3
MSH4
REC8
RFA2
REC104
REC114
MND2
RAD1
YLR445W_1
MCM21
SMC3
TOP2
HOP2
NDJ1
REC107
DMC1
YGL250W
SLX4
MSH2
Gene
Z_Shift_K_Doxo_HLD
Z_lm_K_Doxo_HLD
Z_Shift_K_Doxo_EG
Z_lm_K_Doxo_EG
Z_Shift_L_Doxo_HLD
Z_lm_L_Doxo_HLD
Z_Shift_L_Doxo_EG
Z_lm_L_Doxo_EG
